# Maternal awareness and practices in managing screen-time for children

**DOI:** 10.1192/j.eurpsy.2024.738

**Published:** 2024-08-27

**Authors:** H. Atturu, S. C. Gujju

**Affiliations:** ^1^Dept of Psychiatry, Care Hospital Hitech, Hyderabad, India; ^2^Year 13 A-Level, The English College, Dubai, United Arab Emirates

## Abstract

**Introduction:**

The issue of screen time usage among children has become a contentious topic for parents in contemporary society. While electronic devices offer undeniable benefits, their inappropriate use can lead to substantial mental and physical health challenges for children. Parents are tasked with the responsibility of equipping themselves and their children with the knowledge and skills necessary for mindful electronic device use.

**Objectives:**

This study aims to assess the awareness levels of mothers regarding their children’s screen time usage and to promote mindful screen usage. It also aims to understand the reasons behind parents’ decisions to allow their children access to electronic devices.

**Methods:**

An adapted short online screen-time questionnaire (Vizcaino et al 2019), was distributed through online Google forms, primarily to mothers residing in India. The questionnaire comprised of ten questions encompassing topics related to the child’s background, mothers’ awareness and patterns of screen-time usage.

**Results:**

213 mothers with children aged one year to 17 years responded. 157 mothers (73.7%) were in employment (104 were working in an office, 32 were working from home and 18 were freelancing). 121 mothers had >one child. Majority of the mothers (n=170, 79.81%), believed that children should have <one hour screen-time. However, they also admitted that majority of their children spent >one hour per day screen-time. The usage was more during weekends (>one hour=161, 75.58%) than weekdays (>one hour = 145, 68%)(p=0.021). Weekend screen-time was more in children whose mothers were employed (p=0.006). There is a significant increase in weekday (p=0.044) and weekend (p=0.006) screen-time usage as the child’s age increased. Children predominantly watched television, followed by mobile phones and tablets. Except for 29 children, the rest enjoyed interacting with other people (54 with everyone, 73 with only family members and 57 only for some time). The primary context in which children engaged with electronic devices was while they were being fed/ meals-time (n = 114,54%) or when the mothers were busy with household chores (n = 85,40%).

**Conclusions:**

Despite maternal awareness about healthy screen-time, majority of the children were allowed to use higher screen-time. Efficient strategies should be imparted to parents to change the current practices of using digital-media as pacifier or distractor to mindful screen-time including usage for educational purposes.

**Disclosure of Interest:**

H. Atturu Consultant of: Advisor to CognitiveBotics, AI based software., S. Gujju: None Declared

